# Activation of Human Stearoyl-Coenzyme A Desaturase 1 Contributes to the Lipogenic Effect of PXR in HepG2 Cells

**DOI:** 10.1371/journal.pone.0067959

**Published:** 2013-07-09

**Authors:** Jun Zhang, Yijuan Wei, Bingfang Hu, Min Huang, Wen Xie, Yonggong Zhai

**Affiliations:** 1 Beijing Key Laboratory of Gene Resource and Molecular Development, College of Life Sciences, Beijing Normal University, Beijing, China; 2 Center for Pharmacogenetics, University of Pittsburgh, Pittsburgh, Pennsylvania, United States of America; 3 Institute of Clinical Pharmacology, Sun Yat-Sen University, Guangzhou, Guangdong, China; 4 Key Laboratory for Cell Proliferation and Regulation Biology of Ministry of Education, College of Life Sciences, Beijing Normal University, Beijing, China; Nihon University School of Medicine, Japan

## Abstract

The pregnane X receptor (PXR) was previously known as a xenobiotic receptor. Several recent studies suggested that PXR also played an important role in lipid homeostasis but the underlying mechanism remains to be clearly defined. In this study, we found that rifampicin, an agonist of human PXR, induced lipid accumulation in HepG2 cells. Lipid analysis showed the total cholesterol level increased. However, the free cholesterol and triglyceride levels were not changed. Treatment of HepG2 cells with rifampicin induced the expression of the free fatty acid transporter CD36 and ABCG1, as well as several lipogenic enzymes, including stearoyl-CoA desaturase-1 (SCD1), long chain free fatty acid elongase (FAE), and lecithin-cholesterol acyltransferase (LCAT), while the expression of acyl:cholesterol acetyltransferase(ACAT1) was not affected. Moreover, in PXR over-expressing HepG2 cells (HepG2-PXR), the SCD1 expression was significantly higher than in HepG2-Vector cells, even in the absence of rifampicin. Down-regulation of PXR by shRNA abolished the rifampicin-induced SCD1 gene expression in HepG2 cells. Promoter analysis showed that the human SCD1 gene promoter is activated by PXR and a novel DR-7 type PXR response element (PXRE) response element was located at -338 bp of the SCD1 gene promoter. Taken together, these results indicated that PXR activation promoted lipid synthesis in HepG2 cells and SCD1 is a novel PXR target gene.

## Introduction

Lipid homeostasis is tightly maintained by balanced lipogenesis, catabolism (β-oxidation), and uptake/secretion. Disruptions of lipid formation and catabolism have been implicated in various metabolic diseases, such as obesity and diabetes. Liver is a major organ for lipogenesis, where most lipogenic genes, including the fatty acid synthase (FAS), stearoyl-CoA desaturase-1 (SCD1) and long chain free fatty acid elongase (FAE), are highly expressed. Several nuclear receptors have been implicated in lipid homeostasis, such as the liver X receptors (LXRs) [1], thyroid hormone receptor (TR) [2] and peroxisome proliferator-activated receptors (PPARs). Both LXRα and LXRβ have been shown to promote lipogenesis though direct and indirect mechanism [1], [3], [4]. Upon activation, LXRs form a heterodimer with retinoid X receptor (RXR) and bind to its direct target lipogenic genes promoter, such as FAS, or up-regulate the sterol regulatory element binding protein (SREBP)-1c, a transcriptional factor known to regulate the expression of a battery of lipogenic enzymes [5], [6], [7]. TR can be activated by thyroid hormone and subsequently increase transcription of several genes involved in lipogenesis [8], [9]. PPARs have distinct roles in lipid metabolism. PPARα enhances the metabolic usage of fatty acids by inducing enzymes involved in β-oxidation [10], [11]. PPARγ is a key regulator of adipocyte differentiation and promotes lipid storage in mature adipocytes [12], [13]. Overexpression of PPARγ in liver of PPARα null mice induced the expression of lipogenic genes, leading to hepatic steatosis [14]. CD36, a membrane receptor capable of uptaking modified forms of low-density lipoproteins (LDL) and fatty acids from circulation [15], [16], has been identified as a direct target of PPARγ in liver [17]. While expression of an activated form of PPARδ in the adipose tissues of transgenic mice was shown to activate fat metabolism and produce lean mice that are resistant to obesity induced either genetically or by a high fat diet [18].

The nuclear receptor pregnane X receptor (PXR; NR1I2), originally isolated as a xenobiotic receptor, is highly expressed in the liver, and plays a major role in drug metabolism and elimination through its regulation of the expression of cytochrome P450 enzymes [19]. Several recent studies suggested that PXR is also involved in hepatic lipid homeostasis. Activation of PXR perturbs lipid homeostasis in mice by decreasing β-oxidation, increasing free acid uptake and lipogenesis, which results in hepatic steatosis in mice [20], [21], [22], [23]. Activation of PXR also decreases the expression of carnitine palmitoyltransferase 1A (CPT1A), which controls the entry of activated long-chain fatty acids into the mitochondria, and mitochondrial 3-hydroxy-3-methyl-glutarate-CoA synthase 2 (Hmgcs2), the rate-limiting enzyme of ketogenesis. PXR regulates CPT1A and Hmgcs2 expression through its crosstalk with the insulin-responsive forkhead factor A2 (FoxA2) [21]. Another study showed that in VP-hPXR transgenic mice, the expression of several genes involved in fatty acid β-oxidation, such as PPARα and thiolase, was suppressed [23]. The fatty acid translocase CD36 was established as a direct target gene of PXR. PXR binds to a DR3 type PXRE in the CD36 gene promoter and induces the expression of CD36, increasing the fatty acid uptake in liver [23]. In human hepatocytes (HHPC), PXR activation by rifampicin, a well-known hPXR agonist, stimulates *de novo* lipogenesis through the activation of S14, a small acidic protein that plays an important role in the induction of lipogenic enzymes [20].

Stearoyl-CoA desaturase-1 (SCD1) is the rate-limiting enzyme that converts palmitoyl- and stearoyl-coenzyme A to palmitoleoyl- and oleoyl-coenzyme A, respectively [24]. The monounsaturated products of SCD1 are preferred substrates for the synthesis of triglycerides, cholesterol esters, and phospholipids [24]. The expression of SCD1 is regulated by a number of dietary, physiological and hormonal factors including insulin, fructose, glucose, cholesterol and polyunsaturated fatty acids [25]. Activation of several nuclear receptors, such as LXRs [26], TR [27] and PPARα [28], can induce SCD1 gene expression. SCD1 has been reported as a direct target gene of LXRα and SREBP-1c [29]. In mouse models, activation of PXR induced SCD1 gene expression in the liver. However, whether SCD1 is up-regulated upon PXR activation in human hepatocytes and whether the human SCD1 is a direct PXR target gene are still unknown.

In this study, we showed that activation of PXR in the human hepatoma HepG2 cells induced the expression of SCD1. We also showed that SCD1 is a direct PXR target gene.

## Materials and Methods

### Reagents

Rifampicin, Oil Red O, Isopropanol, 4′,6-diamidino-2-phenylindole (DAPI), TO901317, penicillin and streptomycin were purchased from Sigma (St. Louis, MO). Dimethyl sulfoxide (DMSO) was purchased from Merck (Darmstadt, Germany). Trizol, Dulbecco’s modified Eagle’s medium (DMEM) and fetal bovine serum (FBS) were purchased from Gibco-BRL (Grand Island, NY). BCA-100 protein quantitative analysis kit was from Pierce (Rockford, IL). Rabbit polyclonal anti-PXR (H-160, sc-25381), rabbit polyclonal anti-SCD1 (H-300, sc-30081), rabbit anti-β-actin (sc-1618), goat anti-rabbit IgG-HRP (ZB-2308) were purchased from Santa Cruz Biotechnology (Santa Cruz, CA). Protein molecular weight markers were obtained from Pharmacia (Saclay, France). Polyvinylidene Fluoride (PVDF) membrane for western blotting was obtained from Millipore (Bedford, MA). All other reagents and chemicals were of the highest purity grade available.

### Plasmids

pVP-PXR, pCYP3A4-Luc, pRL-tk and short hairpin RNA (shRNA) construct against the hPXR were kindly provided by Dr. Wen Xie (University of Pittsburg). pCMV-3Xflag was kindly provided by Dr. Richard G. Pestell (Georgetown University). The cDNA of human PXR was subcloned into pCMV-3Xflag between HindIII and XbaI sites by PCR using the following pair of oligonucleotides: forward primer, 5′- ATTAAGCTTCTGGAGGTGAGACCCAAAGA-3′, reverse primer: 5′- ATTTCTAGATCAGCTACCTGTGATGCCGA-3′. The pVP-PXR was used as the PCR template. The different lengths of the 5′-regulatory sequences of human SCD1 gene were cloned by PCR. The forward primers were: 5′- GGAAGATCTATGGTAAGGCTCCTACAGACA-3′ for SCD1-1039, 5′- GGAAGATCTACGGTTTCCACAAAGAAGAT-3′ for SCD1-653, 5′- AATAGATCTGGGCAGAGCCATTGTTCG-3′ for SCD1-436 and 5′- AATAGATCTCGAGGGTTCACCACTGTTT-3′ for SCD1-267. The common reverse primer is 5′- CCCAAGCTTAAATGCTAATGAGGCTTCTG-3′. Genomic DNA isolated from the HepG2 cells was used as the PCR template. The PCR products were cloned into the pGL3 vector between the BglII and HindIII sites. Site-directed mutagenesis was performed by the PCR overextension method [19]. All newly constructed plasmids, as well as the site-directed mutagenesis, were confirmed by DNA sequencing.

### Cell Culture, PXR Stable Cell Line and PXR Knockdown Experiments

HEK293T and HepG2 cells were obtained from the Institute & Hospital Chinese Academy of Medical Sciences. Both cell lines were maintained in DMEM supplemented with 10% FBS, 100 units/ml penicillin and 100 mg/ml streptomycin, and incubated at 37°C in 5% CO_2_. To generate PXR stable cell line, pCMV-3Xflag vector and pCMV-3Xflag-PXR were transfected into HepG2 cells using Lipofectamine from Invitrogen (Carlsbad, CA, USA) according to the manufacturer’s protocol. After 48 h transfection, cells were subcultured (1∶10) into 24-well-plate and then selected by G418 (600 µg/ml) for 14 days. The cell colonies were expanded and the expression of PXR was verified by RT-PCR, western blot and immunostaining. The two stable cell lines were named HepG2-Vector and HepG2-PXR, respectively. For PXR RNA interference experiment, short hairpin RNA (shRNA) constructs against the hPXR in the retroviral pRS backbone were transfected into HepG2 cells using Lipofectamine 2000. Cells were maintained in culture medium for 24 h before rifampicin treatment.

### Oil Red O Staining

HepG2 cells were seeded in 6-well plates and treated with TO901317 or rifampicin for 48 h at indicated concentrations. For Oil Red O staining, cells were washed three times with phosphate-buffered saline (PBS) and fixed with 4% formaldehyde for 1 h. Oil Red O (0.5% in isopropanol), diluted with water (3∶2), filtered twice with a 0.45 µm filter, and added to fixed cells for 15 min at room temperature. Cells were washed with 70% ethanol and water before being visualized by light microscopy and photographed. Then the stained lipid droplets were dissolved in isopropanol and quantified by measuring the absorbance at 510 nm.

### Lipid Profile Analysis

HepG2 cells were treated as described above. The triglyceride and cholesterol levels of the cells were measured using triglyceride assay kit and cholesterol assay kit purchased from Applygen Technologies Inc (Beijing, China), respectively. The cell lysate protein concentrations were measured using the BCA method.

### RNA Isolation and Semi-quantitative RT-PCR

Total RNA was isolated using Trizol reagent (GIBCO-BRL). RNA concentrations were determined using Gene Quant (Amersham Pharmacia Biotech). 2 µg of total RNA were used to synthesize the first strand cDNA. Relative genes expression was determined by semi-quantitative RT-PCR using the Gene Amplify PCR System. The amplification product electrophoresis was carried out on 1.2% agarose gels, visualized by ethidium bromide staining, and photographed. The bands intensity was analyzed using ImageJ from at least three independent experiments. The SCD1 gene mRNA level was confirmed by real-time RT-PCR.

### Western Blot Analysis

Cells were harvested in cell lysis buffer (1×PBS, 1% Nonidet P-40, 0.5% sodium deoxycholate, and 0.1% SDS) containing freshly added protease inhibitor cocktail from Sigma (St. Louis, MO). 20 µg total protein was used to load each well. Proteins were electrophoretically transferred onto PVDF membranes and then blocked in 5% non-fat milk in TBST. The blocked membranes were incubated with anti-PXR, anti-SCD1 or anti-β-actin antibodies (1∶1000) overnight at 4°C and then with secondary antibody (1∶5000) for 2 h at RT. Blots were washed three times with TBST and subsequently developed using the Pierce ECL kit.

### Transient Transfections and Luciferase Activity Assay

HEK293T cells in 24-well plates were transiently transfected with 1.25 µg of total DNA (expression plasmids for 900 ng of PXR, 300 ng of luciferase reporter vector, and 50 ng of pRL-tk) using SuperFect Transfection Reagent from Qiagen (Valencia, CA). 12 h after transfection, cells were treated with rifampicin (10 µM) or DMSO for 24 h. The firefly luciferase luminescence was normalized with the renilla using the Dual luciferase assay system (Promega). Transfection experiments were performed in triplicate and repeated independently for at least three times.

### Immunochemistry Staining

For HepG2-Vector and HepG2-PXR cells, cells seeded in 24-well plates containing a glass cover slip were washed with 1X PBS and then fixed with 4% neutral buffered formaldehyde solution for 20 min at RT. Cells were then treated with 0.1% Triton X-100 in PBS for 10 min at RT. After being blocked with 1% BSA/PBS for 1 h at RT, cells were incubated with anti-PXR antibody (1∶200 in 1% BSA/PBS) overnight at 4°C. After washing with PBS, FITC-tagged goat-anti-rabbit IgG (1∶200 in 1% BSA/PBS) were added to the cells and incubated at RT for 40 min. Then the slides were washed in PBS and mounted on mounting medium containing DAPI. The results were visualized on a Zesis fluorescence microscope. For HEK293T cells, pCMV-3Xflag-PXR were transiently transfected cells in 24-well plates containing a glass cover slip. After 24 h of transfection, cells were treated with rifampicin (10 µM) or DMSO for 2 h. The subsequent procedures were the same as described for the HepG2 cells.

### Electrophoretic Mobility Shift Assay (EMSA)

PXR and RXR receptors proteins were prepared using the TNT *in vitro* transcription and translation system (Promega). The binding reactions were as previously described [30]. Protein-DNA complex was resolved by electrophoresis through 5% polyacrylamide gel in 0.5×TBE at 4°C for 1-3 h. For oligonucleotide competition experiments, unlabeled oligonucleotides were added to the reaction at 100-fold molar excess to the radio-labeled probe.

### Statistics

The intensity of bands in RT-PCR and western blot were analyzed by ImageJ. All data were analyzed by comparing means with one-way ANOVA method using SPSS (Chicago, IL, USA, version 16.0). Data were shown as mean ± SEM and P<0.05 denotes statistically significant difference.

## Results

### Rifampicin Induced PXR Translocation to the Nucleus

**Figure 1:**
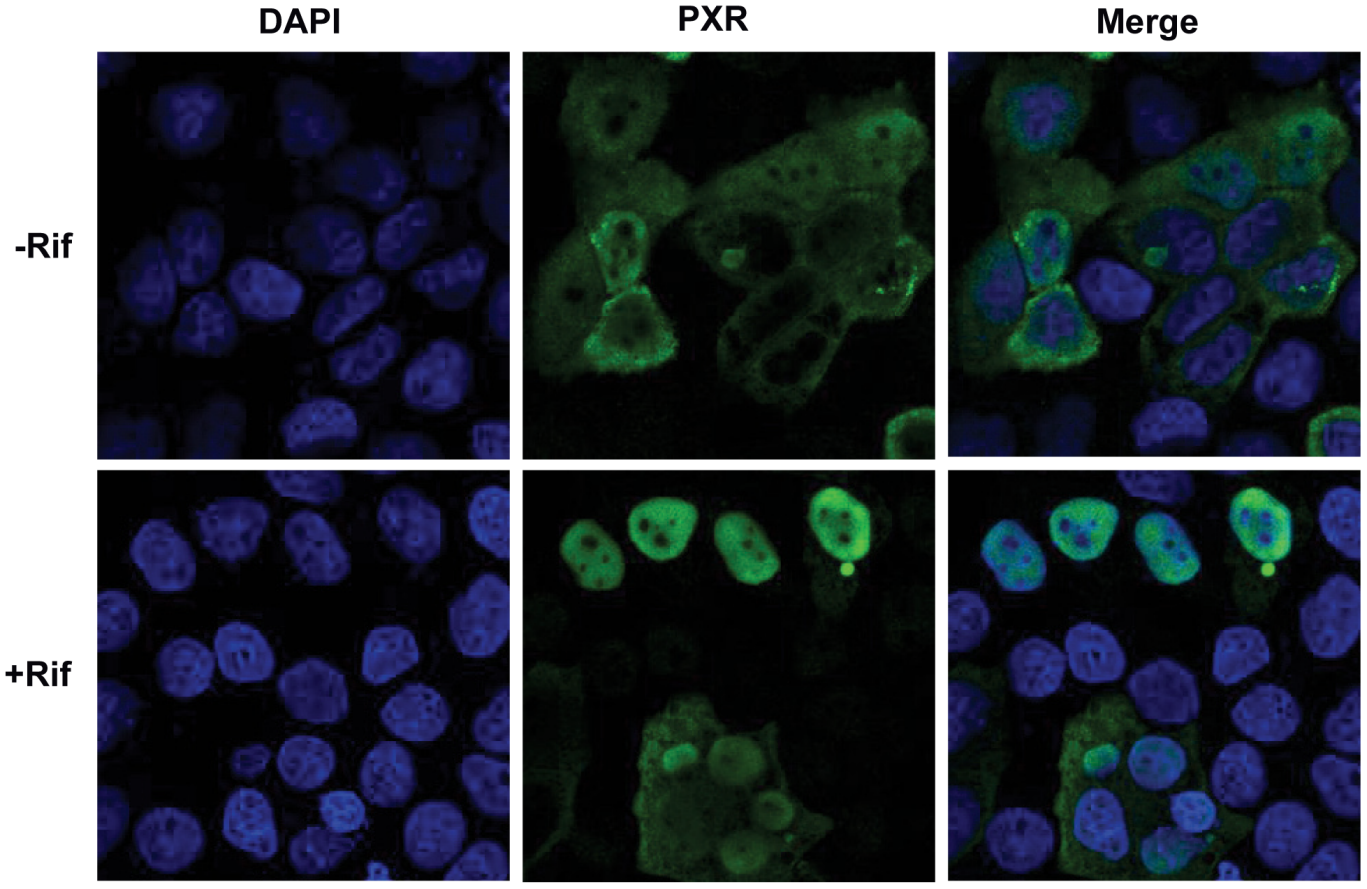
Rifampicin induced PXR nuclear translocation. HEK293T cells were transfected with pCMV-3×flag-hPXR for 24 h, then treated with rifampicin (Rif, 20 µM) for 2 h. PXR was detected using an anti-PXR polyclone antibody and FITC-tagged second antibody. Nucleuses were stained by DAPI.

PXR, known as a xenobiotic receptor, is a ligand-dependent nuclear receptor, which forms heterodimers with the nuclear receptor retinoic X receptor (RXR) upon activation by its agonists and subsequently induces its target genes expression. In this experiment, PXR was transiently transfected into HEK293T cells. Twenty-four hour after transfection, cells were incubated with rifampicin or DMSO for another 2 h. Most of the transfected PXR was located in the cytosol in the absence of ligand (**Figure 1**). Upon rifampicin incubation, PXR translocated to the nucleus (**Figure 1**).

### Rifampicin Induced Lipid Accumulation in HepG2 Cells

**Figure 2:**
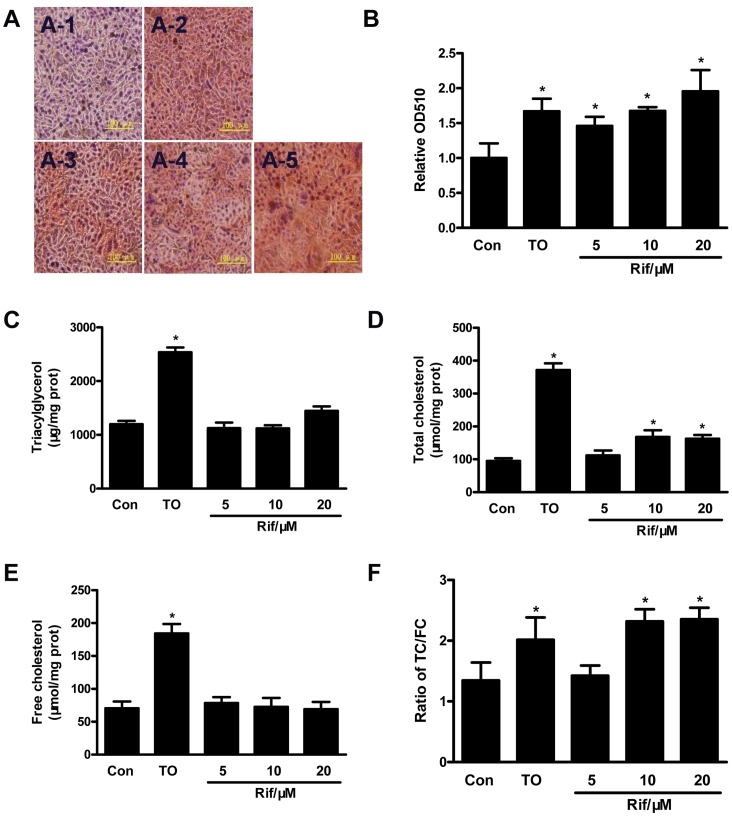
Rifampicin induced lipid accumulation in HepG2 cells. **A.** Oil red O staining of HepG2 cells. HepG2 cells were treated with rifampicin 5 µM (**A-3**), 10 µM (**A-4**), 20 µM(**A-5**) or TO901317 (10 µM, **A-2**) for 48 h. Cells treated with DMSO (**A-1**) were used as control. **B.** The stained lipid content was quantified by measuring absorbance at 510 nm. The triglyceride (TG, **C**), total cholesterol (TC, **D**) and free cholesterol (FC, **E**) levels were measured. **F.** The ratio of TC/FC. All experiments were repeated at least three times. *, P<0.05.

Previous studies have shown that activation of PXR in mice induced hepatic lipid accumulation and steatosis [23]. In order to determine whether activation of the human PXR in human liver cells has the same effect, we treated HepG2 cells with rifampicin. Oil red O staining showed lipid accumulation in HepG2 cells after rifampicin incubation (**Figure 2A** and **Figure 2B**). Lipid profile analysis showed that the triglyceride level was not changed in the cells (**Figure 2C**), whereas the total cholesterol level was significantly increased (**Figure 2D**). However, the free cholesterol level was not changed (**Figure 2E**), indicating that the cholesterol ester level was induced in HepG2 cells after rifampicin treatment.

### Rifampicin Affected the Expression of Genes that Impact Lipid Homeostasis

**Figure 3:**
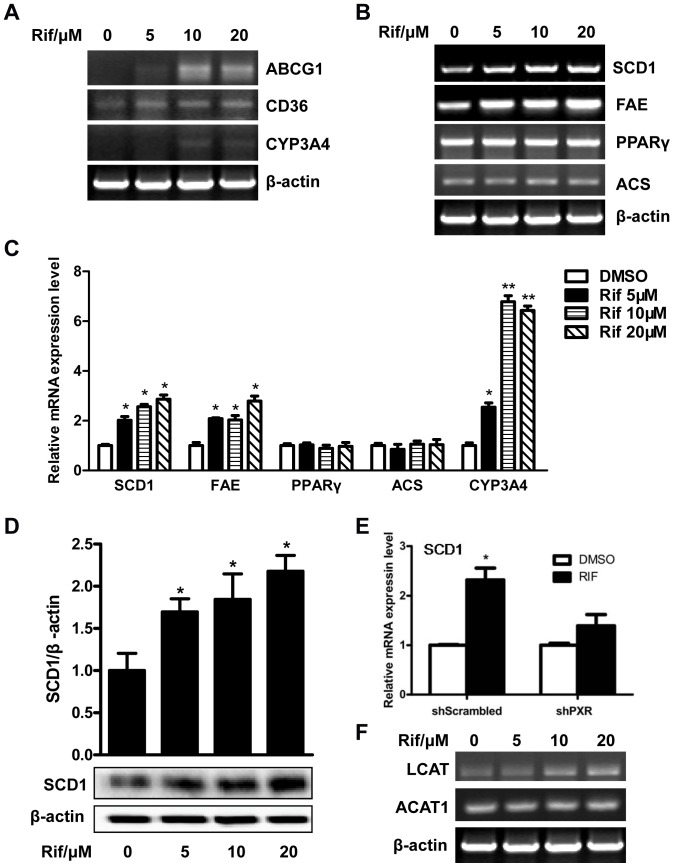
Genes expression analysis in HepG2 cells. HepG2 cells were treated with rifampicin at indicated concentrations for 48 h. Total RNA was isolated and the selected lipid metabolism genes expression was determined by RT-PCR. **A.** Expression of CYP3A4, CD36 and ABCG1. **B.** Expression of several lipogenic genes. **C**, The relative gene level was analyzed using ImageJ from at least three independent experiments. *, P<0.05; **, P<0.01. **D**, Knockdown of PXR by shRNA abolished rifampicin-induced SCD1 gene expression in HepG2 cells. **E.** The SCD1 gene protein level in HepG2 cells after incubation with rifampicin. The intensity of the bands was measured using ImageJ. *, P<0.05. **F.** The expression of LCAT and ACAT1 gene.

The lipid accumulation in HepG2 cells prompted us to examine the effect of rifampicin on the expression of genes that affect lipid homeostasis. RT-PCR analyses showed that the expression of CD36, a fatty acid translocase that is responsible for the high affinity uptake of fatty acids, was up-regulated (**Figure 3A**). In mouse models, CD36 has been established as a common target gene of LXR, PXR and PPARγ in promoting steatosis [31]. The expression of ATP-binding cassette sub-family G member 1 (ABCG1), a gene involved in cholesterol and phospholipids transport, was also increased (**Figure 3A**). The expression of PPARγ was not affected (**Figure 3B**), which was different from the results from mice [23], [30]. In addition, the expression of two lipogenic enzymes, stearoyl-CoA desaturase-1 (SCD1) and long chain free fatty acid elongase (FAE) (**Figure 3B**), was induced. The relative expression of several genes was analyzed using ImageJ from at least three independent experiments (**Figure 3C**). The protein level of SCD1 was also increased as shown by western blot analysis (**Figure 3D**). Down-regulation of PXR by shRNA abolished rifampicin-induced SCD1 gene expression in HepG2 cells (**Figure 3E**). The design and efficiency of PXR knockdown by shRNA has previously been validated [32]. Interestingly, the expression of lecithin-cholesterol acyltransferase (LCAT) was increased (**Figure 3F**), which was consistent with the change of cholesterol ester level in rifampicin-treated HepG2 cells. However, the expression of ACAT1(acyl:cholesterol acetyltransferase), an enzyme that catalyzes esterification of free cholesterol and fatty acids in hepatocytes, was not affected by rifampicin in HepG2 cells (**Figure 3F**). CYP3A4, a known PXR target gene, was induced as expected (**Figure 3A**).

### Establishment of PXR-overexpressing HepG2 Cells

**Figure 4:**
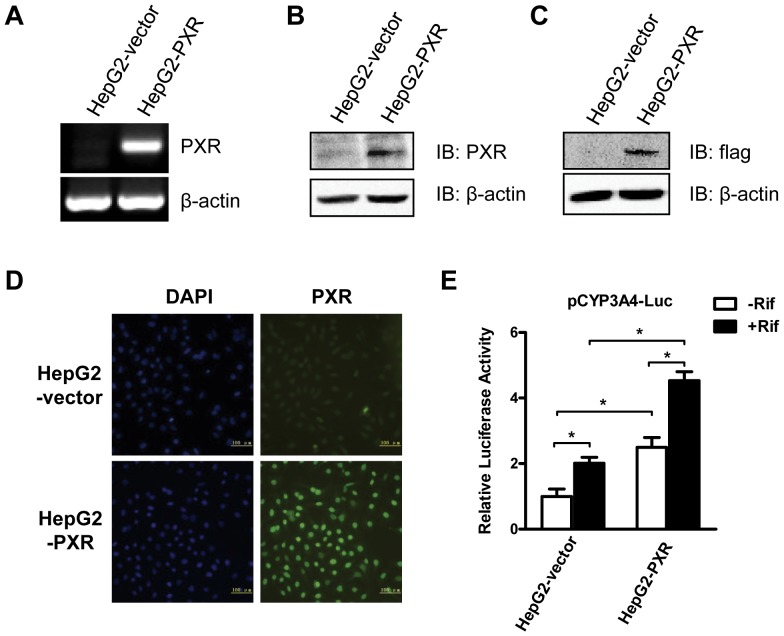
Establishment of the HepG2-PXR stable cell line. The selection methods of HepG2-PXR and HepG2-Vector cells were described in Materials and Methods. **A to D.** The selected cells were verified for PXR expression using RT-PCR (**A**), western blot using an anti-PXR antibody (**B**) and an anti-flag antibody (**C**), and immunofluorescence using an anti-PXR antibody (**D**). **E.** Dual-luciferase assay of transcriptional activity of PXR on CYP3A4 in HepG2-Vector and HepG2-PXR cells. pCYP3A4-Luc and pRL-tk were transfected into these two cell types for 24 h, and then treated with rifampicin (10 µM) for another 24 h. Three independent experiments were performed. *, P<0.05.

**Figure 5:**
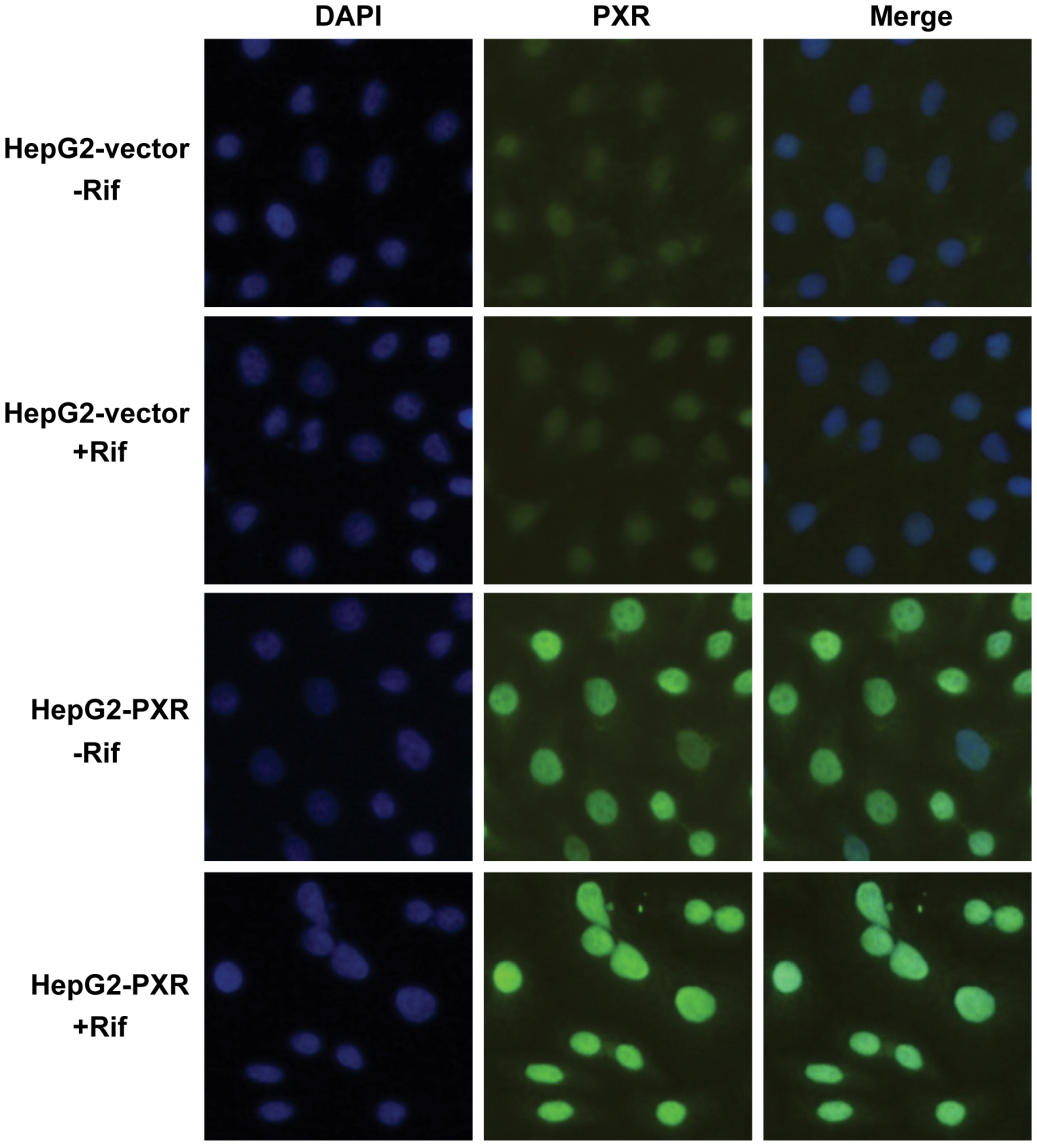
Most overexpressed PXR was located in the nucleus of HepG2-PXR cells. HepG2-PXR and HepG2-Vector cells were seeded in 24-well plates contained a glass coverslips. Cells were then treated with rifampicin for 2 h. Immunofluorescence using an anti-PXR antibody was used to determine the PXR localization. The nucleus was stained by DAPI.

Because of the low level of PXR expression in HepG2 cells, we constructed HepG2-PXR cell line that stably overexpresses PXR in order to better study the effect of PXR on lipogenesis. Human PXR expression plasmid, pCMV-3Xflag-PXR, and control vector plasmid, pCMV-3Xflag, were transfected into HepG2 cells, which were then selected by G418 for 14 days. The cell colonies were selected and expanded. The PXR and vector cell lines were named HepG2-PXR and HepG2-Vector, respectively. The expression of PXR at both mRNA and protein levels was verified. RT-PCR analysis showed that the mRNA level of PXR in HepG2-PXR cells was much higher than in HepG2-Vector cells (**Figure 4A**). The PXR protein expression was confirmed by western blot analysis using an anti-PXR antibody (**Figure 4B**) and an anti-flag antibody (**Figure 4C**), and by immunofluorescence using an anti-PXR antibody (**Figure 4D**). To functionally test the stable cells, pCYP3A4-Luc was transfected into HepG2-PXR and HepG2-Vector cells and the transfected cells were treated by rifampicin. As expected, compared with HepG2-vector cells, the transcriptional activity of PXR on the CYP3A4 promoter reporter gene was significantly higher in HepG2-PXR cells after rifampicin activation (**Figure 4E**). The basal reporter activity in HepG2-PXR cells was also higher than HepG2-Vector cells (**Figure 4E**). These results were consistent with the cellular localization of PXR in HepG2-PXR cells. As shown in immunochemistry staining, even in the absence of rifampicin, most PXR protein was located in the nucleus (**Figure 5**), while in HepG2-Vector cells, PXR was evenly distributed within the cells (**Figure 5**). Upon rifampicin incubation, PXR translocated into the nucleus in both HepG2-Vector and HepG2-PXR cells (**Figure 5**).

### The Expression of SCD1 was Induced in HepG2-PXR Cells

**Figure 6:**
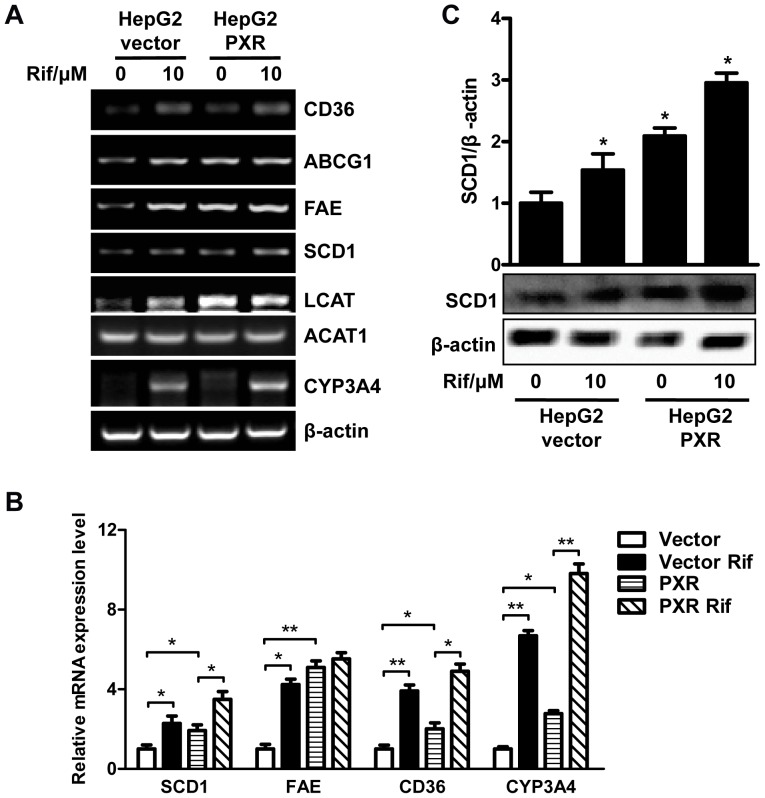
Genes expression in PXR-HepG2 cells. HepG2-PXR and HepG2-Vector cells were treated with rifampicin (10µM) or DMSO for 48 h. **A.** Total RNA was isolated and the selected genes expression was determined by RT-PCR. **B**, The relative gene level was analyzed using ImageJ from at least three independent experiments. *, P<0.05; **, P<0.01. **C.** The protein level of SCD1 in the two type of cells with or without rifampicin treatment was determined by western blot. The bands intensity was measured using ImageJ. *, P<0.05.

We next examined the expression of genes involved in lipid homeostasis in HepG2-PXR and HepG2-Vector cells with or without rifampicin incubation. As expected, the expression of CD36, ABCG1, FAE, SCD1, LCAT and CYP3A4 was increased in both cell lines after rifampicin treatment (**Figure 6A**), which was consistent with the results in the parent HepG2 cells. Moreover, the expression of these genes in HepG2-PXR cells was higher than in HepG2-Vector cells (**Figure 6A**). The relative expression of several genes was analyzed using ImageJ(**Figure 6B**). The protein level of SCD1 was also significantly induced upon rifampicin treatment, which was determined by western blot analysis (**Figure 6C**).

### SCD1 was a Direct Transcriptional Target of PXR

**Figure 7:**
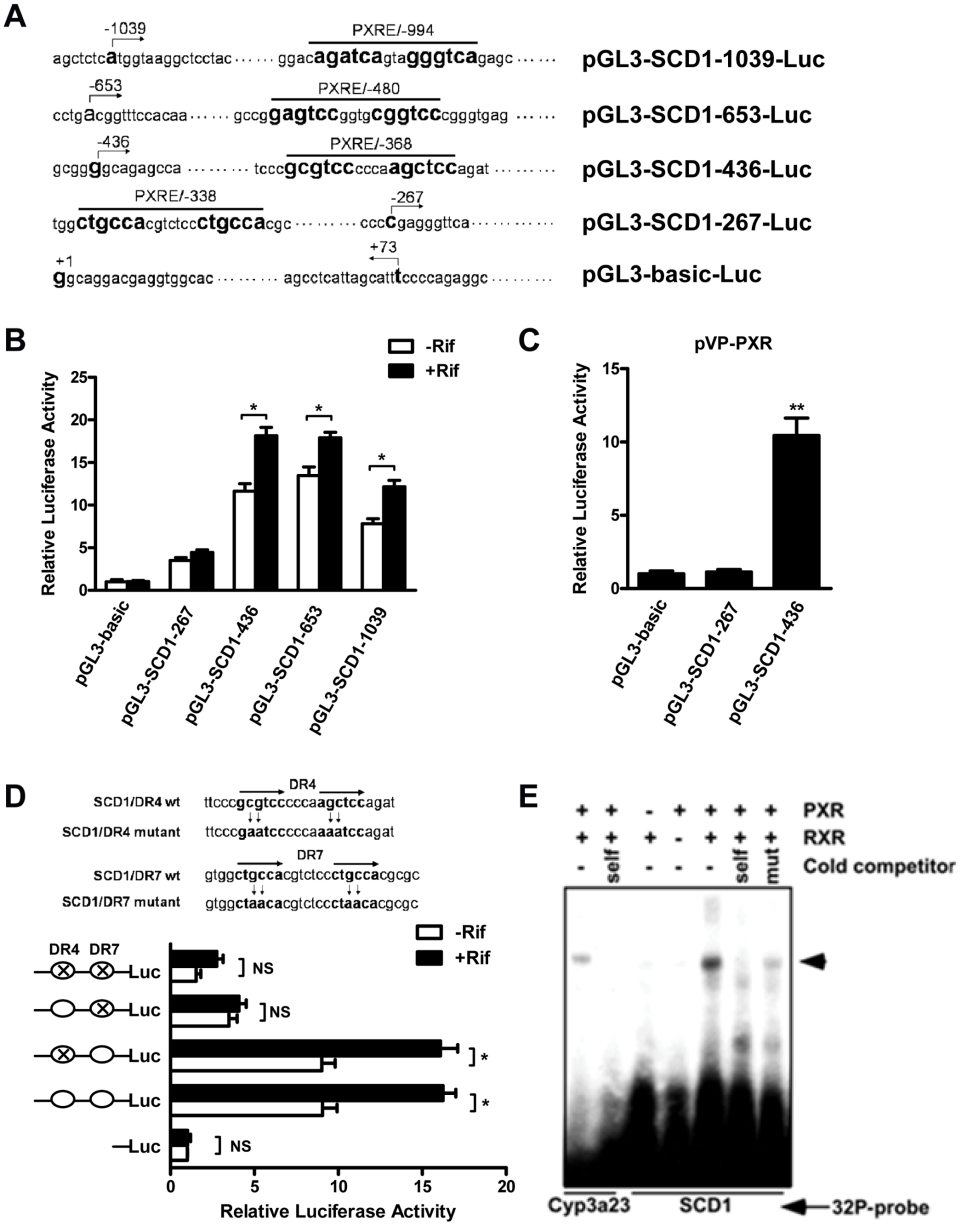
SCD1 is a direct target gene of PXR. **A.** Predicted putative PXREs on the SCD1 gene promoter and luciferase report gene constructs. **B.** The SCD1 promoter luciferase report genes and pRL-tk were co-transfected into HepG2 cells for 24 h, followed 24 h treatment with rifampicin (+Rif) or DMSO (-Rif). The luciferase activities were measured. *, P<0.05. **C.** The SCD1 promoter luciferase report genes and pRL-tk were co-transfected with pVP-PXR expression vector into HepG2 cells for 24 h, and then the luciferase activities were measured. **, P<0.01. **D.** Site-direct mutagenesis of DR4 and DR7 elements on SCD1 promoter and then the transcriptional activity of PXR was measured. *, P<0.05. **E.** The binding of the PXR-RXR heterodimers to DR7 was confirmed by EMSA. The binding of PXR-RXR heterodimers to a DR3 type PXRE from the rat Cyp3a23 gene was included as a positive control. The arrowhead indicates the shift bands.

Based on the results of previous studies which found that SCD1 was induced both in PCN treated mouse liver and hPXR transgenic mouse liver, and our current results that SCD1 was also up-regulated in rifampicin treated HepG2 cells and HepG2-PXR cells, we hypothesized that SCD1 is a direct transcriptional target of PXR. Inspection of the hSCD1 gene promoter revealed several potential PXR response elements (PXREs) (**Figure 7A**). SCD1 promoter report genes containing different lengths of SCD1 gene promoter were constructed (**Figure 7A**). Transient transfections and dual-luciferase reporter gene assays were used to determine whether and which potential elements were necessary and sufficient in mediating the PXR transactivation in HepG2 cells. As shown in **Figure 7B**, the luciferase report gene that contained a fragment from -267 bp to -436 bp from the transcription start site of SCD1 gene was activated by rifampicin. The luciferase report gene was also activated by co-transfection with a plasmid expressing VP-PXR, a constitutively activated PXR (**Figure 7C**). These results indicated that a potential PXRE might exist within this segment. There are two potential PXREs in this region, one is a DR4 type (GCGTCCcccaAGCTCC) located at -368 bp to -353 bp, and the other is a DR7 type (CTGCCAcgtctccCTGCCA) located at -338 bp to -320 bp. We next mutated these two sites and repeated transient transfections and dual-luciferase reporter gene assays. As shown in **Figure 7D**, when only the DR4 element was mutated, the luciferase report gene remained activated by rifampicin. While the reporter activity was abolished when the DR7 element was mutated, indicating that the DR7 element was required in mediating the PXR transactivation. The binding of the PXR-RXR heterodimers to the DR7 element was confirmed by EMSA. As shown in **Figure 7E**, the PXR-RXR heterodimers bound to DR7 efficiently. The binding was specific because the binding can be efficiently competed away by the unlabeled cold probe, but not by the unlabeled mutant probe. The binding of PXR-RXR heterodimers to a DR3 type PXRE from the rat Cyp3a23 gene [19]was included as a positive control.

## Discussion

In this study, we showed that rifampicin induced lipid accumulation in HepG2 cells through the up-regulation of several genes involved in hepatic lipid uptake and lipogenesis, such as the free fatty acid transporter CD36 and lipogenic enzymes FAE and SCD1. We also established SCD1 as a direct transcriptional target of PXR.

PXR overexpression and activation in VP-hPXR transgenic mice caused hepatic steatosis, which is characterized by a marked accumulation of hepatic triglycerides [23]. This is a result from combined effect of PXR activation on increased hepatic free fatty acid uptake, lipogenesis and suppression of β-oxidation [23]. The PXR-mediated lipogenesis in rodents is independent of SREBP-1c, which is distinct from that mediated by LXR [7], [33]. However, the effect of PXR on lipogenesis in human liver cells has not been reported. In the current study, although the triglyceride level in HepG2 cells was not changed by rifampicin (**Figure 2C**), the total cholesterol level was increased (**Figure 2D**), mainly due to the increased cholesterol ester in HepG2 cells (**Figure 2E and 2F**). Consistent with these observations, the expression of LCAT, an enzyme that converts free cholesterol into cholesteryl ester, was up-regulated by rifampicin, though the expression of ACAT1 was not affected (**Figure 3F** **and** **Figure 6A**). It remains to be determined whether LCAT is a direct target gene of PXR.

SCD1 is a δ-9 desaturase and is the rate-limiting enzyme responsible for converting palmitic (16∶0) and stearic acid (18∶0) to palmitoleic (16∶1) and oleic (18∶1) acids, respectively [24]. SCD1 gene expression is altered by a remarkable number of nutrients, hormones and environmental factors [24], [25]. Several nuclear receptors, such as LXRs [26], TR [27] and PPARα [28] were involved in the regulation of SCD1. SCD1 expression is positively regulated by LXR, either directly through the binding of LXR to an LXR response element in the SCD1 gene promoter, or indirectly through LXR-mediated activation of SREBP-1c transcription [34], [35], [36]. Dietary carbohydrates can increase hepatic SCD1 gene expression through both SREBP-1c-dependent and independent mechanisms [37]. Activation of the cellular immune response via toll-like receptor 2 also increases the transcription of SCD1, potentially via the nuclear factor κB elements in the SCD1 gene promoter [38].

Our transcription factor binding sites screen on the human SCD1 gene promoter indicated several potential PXR binding elements (PXREs) in the 2000-bp region of upstream of the transcription start site (**Figure 7A**). Our promoter analysis results have provided evidence to support SCD1 as a direct transcriptional target of PXR. Both pharmacological (Rifampicin) and genetic (VP-PXR) activation of PXR were sufficient to induce SCD1 expression (**Figure 7B and 7C**). A DR7 type element (CTGCCAcgtctccCTGCCA) at the -338 bp to -320 bp of SCD1 promoter was identified as a PXRE, where PXR directly bound to this element and activated SCD1 transcription upon activation. To our knowledge, this is the first report that PXR can bind to a DR7 type of PXRE. PXR has been reported to bind to DR4 type (CYP3As [39] and S14 [20]), ER6 type (CYP3A4), and DR3 type (CD36 [23]) of PXREs.

In summary, we showed that PXR activation in the human hepatoma HepG2 cells induced lipid accumulation though up-regulation of several hepatic lipogenic genes. The human SCD1 was identified to be a direct transcriptional target of PXR via a novel DR7 type PXRE on the SCD1 gene promoter.
